# INCORPORATING GEOGRAPHIC INFORMATION SYSTEMS (GIS) MAPPING INTO GERONTOLOGY RESEARCH AND TEACHING

**DOI:** 10.1093/geroni/igae098.1392

**Published:** 2024-12-31

**Authors:** Taylor Jansen, Chae Man Lee, Nina Silverstein, Elizabeth Dugan

**Affiliations:** University of Massachusetts Boston, Boston, Massachusetts, United States; University of Connecticut, Farmington, Connecticut, United States; University of Massachusetts Boston, Needham, Massachusetts, United States; University of Massachusetts Boston, Boston, Massachusetts, United States

## Abstract

In gerontology, research frameworks like the life course perspective and social determinants of health (SDOH) are often used to contextualize aging and health outcomes in later life. Increasingly, an emphasis on data visualization has been put on research to have practical value. Geographic information systems (GIS), a mapping software, is one data visualization tool researchers can utilize in their work to understand what role place, space, and built environment have on health throughout the lifespan and in later life. Incorporating GIS training into gerontology research allows for students to learn data visualization, but also to apply their teachings of the life course perspective and SDOH to further understand the impact on health in later life. In this presentation, the aims are twofold: 1) explain the practical uses of GIS in gerontological research, with examples from recent work by the Healthy Aging Data Reports, and 2) describe the process of engaging undergraduate and graduate students in research, teaching, service, and policy through GIS mapping. Firstly, examples of how GIS mapping has provided communities and states with information on their aging populations will be examined. Examples of how community stakeholders in New England, Mississippi, and Wyoming have used maps to implement interventions and advocate for policy change will be described to demonstrate the practical implications of mapping for policy change. Then, best practices of teaching and training undergraduate and graduate students on using GIS mapping for gerontological research will be offered, alongside anecdotal evaluations from students on the experience.

